# Relationship between HgbA1c and Myocardial Blood Flow Reserve in Patients with Type 2 Diabetes Mellitus: Noninvasive Assessment Using Real-Time Myocardial Perfusion Echocardiography

**DOI:** 10.1155/2014/243518

**Published:** 2014-07-02

**Authors:** Runqing Huang, Sahar S. Abdelmoneim, Lara F. Nhola, Sharon L. Mulvagh

**Affiliations:** ^1^Mayo Clinic Cardiovascular Ultrasound and Hemodynamic Laboratory, Division of Cardiovascular Diseases and Internal Medicine, Mayo Clinic, 200 First Street SW, Rochester, MN 55905, USA; ^2^Division of Ultrasound, Tongji Hospital, Tongji Medical College, Huazhong University of Science and Technology, Wuhan 430030, China; ^3^Division of Cardiovascular Medicine, Assiut University, Assiut 71515, Egypt

## Abstract

To study the relationship between glycosylated hemoglobin (HgbA1c) and myocardial perfusion in type 2 diabetes mellitus (T2DM) patients, we prospectively enrolled 24 patients with known or suspected coronary artery disease (CAD) who underwent adenosine stress by real-time myocardial perfusion echocardiography (RTMPE). HgbA1c was measured at time of RTMPE. Microbubble velocity (*β* min^−1^), myocardial blood flow (MBF, mL/min/g), and myocardial blood flow reserve (MBFR) were quantified. Quantitative MCE analysis was feasible in all patients (272/384 segments, 71%). Those with HgbA1c > 7.1% had significantly lower *β*_reserve_ and MBFR than those with HgbA1c ≤ 7.1% (*P* < 0.05). In patients with suspected CAD, there was a significant inverse correlation between MBFR and HgbA1c (*r* = −0.279, *P* = 0.01); however, in those with known CAD, this relationship was not significant (*r* = −0.117, *P* = 0.129). Using a MBFR cutoff value > 2 as normal, HgbA1c > 7.1% significantly increased the risk for abnormal MBFR, (adjusted odds ratio: 1.92, 95% CI: 1.12–3.35, *P* = 0.02). Optimal glycemic control is associated with preservation of MBFR as determined by RTMPE, in T2DM patients at risk for CAD.

## 1. Introduction

Type 2 diabetes mellitus (T2DM) is a known risk factor of coronary artery disease (CAD). Cardiovascular disease is the leading cause of death in T2DM patients. Antecedent to and associated with epicardial coronary artery stenosis, T2DM patients develop abnormal microvascular function in systemic circulatory beds, including those of the myocardium [1–4]. Glycosylated hemoglobin (HgbA1c) has been established as a risk factor for T2DM patients developing microvascular atherosclerosis [5]. However, the relationship between HgbA1c, coronary artery disease (CAD), and coronary perfusion in T2DM patients has not yet been clarified.

HgbA1c level is utilized clinically as an indicator of the adequacy of glycemic control over several months prior to testing. Thus, it is felt to reflect the effectiveness of long-term glucose control in diabetes patients. The American Diabetes Association has recommended that an HgbA1c breakpoint of 7% would realize the greatest cardiovascular benefit [6]. Several studies have shown that HgbA1c is associated with the severity and progression of coronary atherosclerosis [7–9]. The risk of microvascular complications rises exponentially rather than linearly as HgbA1c increases. Conversely, each 1% reduction in HgbA1c has been shown to be associated with a 37% decrease in risk for microvascular complications and a 21% decrease in the risk of any end point or death related to diabetes [10].

The coronary system can be viewed as having two vascular parts [11]. One is composed of the larger epicardial coronary arteries, having diameters of several millimeters (up to 400 *μ*m), and termed “conductive vessels,” with low resistance to blood flow. The coronary arteries then branch into smaller arterioles (resistance vessels), and then branch yet again and again, to end in the smallest branches, comprising the second and largest part of the myocardial circulation, the capillaries, having the smallest diameters (<100 *μ*m). Atherosclerosis in T2DM can occur both within the epicardial and microvascular circulation. However, diffuse microvascular atherosclerosis observed in T2DM is likely far more significant as it affects multiple vascular beds within the body, impacting upon diabetic complications and overall survival. Yet, microvascular disease in T2DM remains difficult to detect and document, generally requiring invasive methodology, and thus is infrequently performed.

Real-time myocardial perfusion echocardiography (RTMPE) is a noninvasive method to evaluate microcirculatory perfusion by depletion (destruction) of microbubbles and their observed replenishment into the myocardium. By videodensitometric analysis of these refill/replenishment cycles, RTMPE can quantitatively measure myocardial perfusion parameters including microbubble velocity *β* (min^−1^), myocardial blood flow (MBF, mL/min/g), and myocardial blood flow reserve (MBFR). We have previously shown that T2DM patients with known or suspected CAD have impaired RTMPE-derived quantitative myocardial perfusion parameters compared to nondiabetic patients during adenosine vasodilator stress [12].

In the current study, our aim was to determine if there was relationship between the HgbA1c level and quantitative myocardial perfusion parameters in T2DM patients with known or suspected CAD, and to determine if the HgbA1c level was an independent risk factor for prediction of myocardial perfusion status.

## 2. Methods

### 2.1. Study Population

We prospectively enrolled 24 T2DM patients (16 male; mean age: 66 ± 12 yrs.) with known or suspected CAD. The mean HgbA1c level was 7.1 ± 1.4% (range 5.4–10.9%), fasting plasma glucose was 151.6 ± 61.2 mg/dL (range 80–289 mg/dL), and duration of diabetes was 8 ± 5.1 years (range 2–25 years). Eleven patients were receiving oral hypoglycemic therapy, twelve were on insulin treatment, and one was on diet control. Patients were classified into 2 groups based on the sampled population mean threshold HgbA1c of 7.1%, which coincided with the recommended goal by the American Diabetes [6]: “Poorly controlled” = Group 1: HgbA1c level > 7.1%, and “Well-controlled” = Group 2: HgbA1c level ≤ 7.1%. Exclusion criteria included age < 18 years, moderate to severe valvular heart disease, congenital heart disease, heart failure, or contraindications to echocardiographic contrast agent or adenosine. The study was approved by the Mayo Clinic Internal Review Board, and all patients gave informed consent.

### 2.2. Imaging Protocol

Rest and stress RTMPE were performed using SONOS 7500 or iE33 (Philips Healthcare, Andover, MA, USA) ultrasound equipment. Definity (Lantheus Medical Imaging; North Billerica, MA, USA) 1.3 mL diluted in 60 cc 0.9% saline was infused continuously at 200 mL/hr. Definity infusion started 1 minute before RTMPE acquisition at rest and was kept constant throughout the study. Stress RTMPE images were continuously acquired after 3 minutes of adenosine infusion (140 *μ*g/kg/min) and completed within 1 minute after discontinuation of the 6-minute adenosine infusion. Apical -4, -3, and -2 chambers and short axis views were acquired using the power modulation setting at a mechanical index (MI) of ≤ 0.2, frame rate of approximately 20 Hz, and transmit focus optimally adjusted at mitral valve level. Depletion-replenishment imaging was used with a transient, high-MI (1.2) to deplete myocardial microbubbles completely (for approximately 10–15 frames), and then replenishment was observed over 15 cardiac cycles. Images were stored digitally for offline analysis.

### 2.3. Image Interpretation

Quantitative RTMPE analysis was performed offline by a single observer (SSA) blinded to patient clinic data and image stage (rest versus stress). Images were evaluated from end-systolic frames using QLAB, version 5.0 (Philips Healthcare, Andover, MA, USA). According to the standard 16-segment myocardial region model, segmental regions of interest (ROI) were placed and tracked manually within the myocardium and in the adjacent left ventricular cavity at end-systolic frames. With regard to the replenishment curve parameters, A (dB) represents the plateau acoustic intensity reflecting microvascular cross-sectional area or myocardial blood volume and *β* (min^−1^) represents the rate of rise of acoustic intensity increase reflecting microbubble velocity; thus, the product *A* × *β* is a semiquantitative estimate of MBF stress [13]. However, myocardial blood volume reflected by *A* is dependent on the ultrasound microbubble agent, scanner settings, and acoustic tissue properties, and it may vary within and between myocardial regions stress [14]. Therefore, we used absolute MBF (mL/min/g) to assess myocardial perfusion. The methodology and terminology for these calculations, described in detail above, and in our previous quantitative and qualitative RTMPE publications and seminal work by Vogel et al. [12, 14], are summarized as follows: absolute MBF = rBV × *β*/*Ρ*t, where rBV (relative blood volume) = *A*/*A*_LV_, *A*_LV_ (dB) is the adjacent left ventricular videointensity in the near wall cavity and *Ρ*t is the myocardial tissue density (1.05 g/mL). Thus, “absolute” MBF = (*A*/*A*_LV_) × *β*/*Ρ*t. Reserve values were calculated as the ratio of hyperemic to baseline values of MBF.

### 2.4. Statistical Analysis

Continuous data were reported as mean ± standard deviation or median (25% IQR, 75% IQR). Frequencies were used to report categorical variables and were compared using the chi-square test or Fisher's exact test accordingly. RTMPE feasibility was evaluated by reporting the percentage of analyzable myocardial segments. Wilcoxon sign rank test was used to compare quantitative RTMPE variables before and after adenosine stress. Wilcoxon rank sum test was used to compare quantitative RTMPE variables between two groups. The correlation between HgbA1c and RTMPE parameters was assessed with Spearman rank correlation. Univariate and multivariate logistic regression analysis was used to evaluate the HgbA1c as a risk factor for decreased MBF relative to traditional risk factors. Variables with likelihood test *P* value < 0.2 in univariate analysis were included in the multivariate logistic regression model and provided final adjusted odds ratios and 95% confidence intervals. In all analyses the significance level was set at two-tailed *P* < 0.05. All analyses were performed using JMP version 9.0 (SAS Campus Drive, Cary, NC, USA).

## 3. Results

### 3.1. Clinical Data

**Figure 1:**
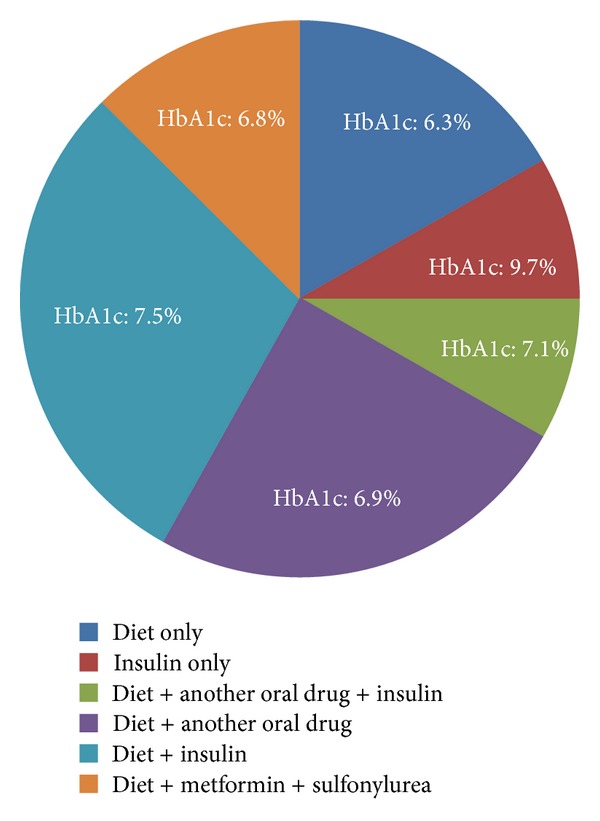
Proportion of study population receiving diabetes treatments and mean HgbA1C for each treatment group.

There were 9 patients in Group 1 (poorly controlled) and 15 patients in Group 2 (well-controlled). Baseline clinical characteristics of the study groups are summarized in Table 1. There were no significant differences in clinical characteristics between two groups. Similarly, hemodynamic recordings at rest or during vasodilator stress did not differ significantly between two groups. Overall, adenosine administration resulted in significant increase (mean ± SE) in both heart rate [10.0 ± 2.1 bpm, (*P* < 0.0001)] and rate-pressure product [876.54 ± 391 bpm × mmHg (*P* < 0.03)]. The majority of patients were taking insulin and/or oral hypoglycemic drugs. The treatment details (% of study population) are shown in Figure 1, along with the mean HgbA1c level for each treatment group.

### 3.2. Quantitative MCE

**Figure 2:**
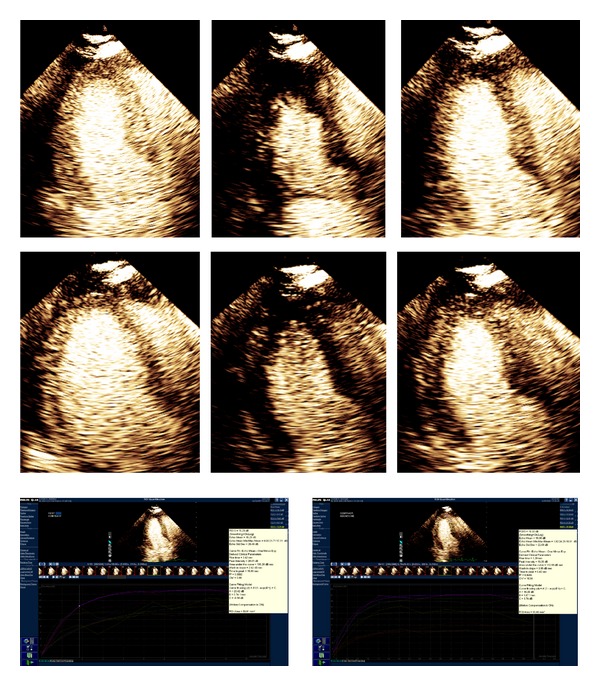
An example of T2DM patient perfusion sequence and analysis curve of 6 segments. 2D imaging of predepletion; depletion (flash), repletion (from left to right; upper: baseline; below: stress), and analysis curve of 6 segments (upper: baseline, below: stress).

Quantitative MCE parameter measurements were feasible in all 24 patients. Of 384 total segments, 272 segments (71%) were analyzable both at rest and peak, in order to derive the MBF reserve value. Inability to perform quantitative MCE analysis was related to failure of curve fitting algorithm or lack of complete transmural visualization of the myocardium (Figure 2).

At rest, there were no differences in *β* between the two groups (*P* = 0.33); however, Group 1 had significantly reduced rBV and MBF compared to Group 2 (*P* < 0.001). After adenosine stress, both groups had significant increases in rBV, *β*, and MBF relative to their resting values (*P* < 0.001). However, after adenosine stress, Group 1 had significantly lower rBV, *β*, and MBF values compared to Group 2 (*P* < 0.001). *β*_reserve_ and MBFR were significantly lower in Group 1 when compared to Group 2 (*P* < 0.001) (Table 2).

**Figure 3:**
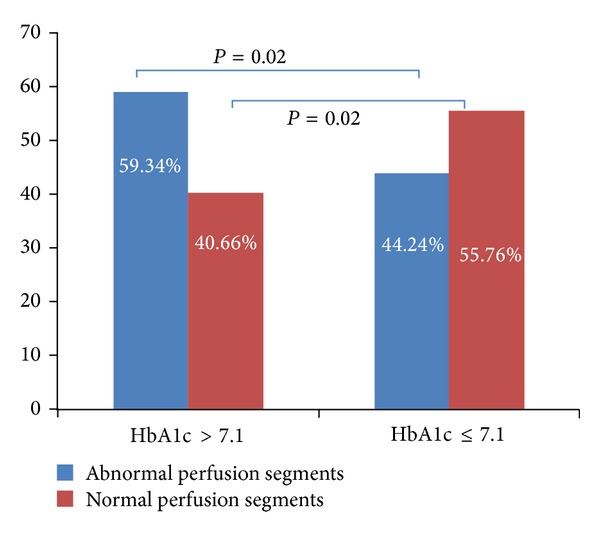
Percent of abnormal and normal perfusion segments compared between two groups.

HgbA1c > 7.1 group has higher proportion of abnormal segments and lower proportion of normal segments than HgbA1c ≤ 7.1 group, *P* < 0.02 (Figure 3).

When patients were stratified according to status of CAD diagnosis (known versus suspected), there was a significant inverse correlation between MBFR and HgbA1c% (*r* = −0.279, *P* = 0.01) in patients with suspected, but not known CAD. However, in those with known CAD, this inverse correlation was present, but not significant (*r* = −0.117, *P* = 0.129).

Using a MBFR cutoff value of >2 as normal, the variables of sex, age, obesity (BMI ≥ 30 kg/m^2^), smoking status, dyslipidemia, duration of T2DM, presence of CAD, and/or hypertension were included in the univariate models, and HgbA1c% > 7.1% significantly increased the risk for having abnormal MBFR (unadjusted odds ratio: 1.84, 95% CI: 1.10–3.11, *P* = 0.02). After further adjustment for variables with likelihood test *P* value < 0.2 in univariate analysis, abnormal MBFR remained significant (adjusted odds ratio: 1.92, 95% CI: 1.12–3.35, *P* = 0.02) (Table 3).

### 3.3. Intra- and Interobserver Variability

For interobserver and variability of quantitative MCE perfusion analysis in our echo lab, the mean differences ± SE and the *r* for rBV reserve, *β* reserve, and MBF reserve were 5.85 ± 0.81 (*r* = 0.371, *P* < 0.001), 1.34 ± 0.04 (*r* = 0.623, *P* < 0.001), and 5.96 ± 0.17 (*r* = 0.544, *P* < 0.001), respectively, while for interobserver variability they were, 4.26 ± 0.61 (*r* = 0.308, *P* < 0.001), 0.57 ± 0.05 (*r* = 0.528, *P* < 0.001), and 1.72 ± 0.06 (*r* = 0.528, *P* < 0.001), respectively.

## 4. Discussion

Impaired coronary flow reserve (CFR) in T2DM patients has been demonstrated in studies using invasive coronary Doppler flow wires stress [15], and noninvasive, but ionizing radioactive nuclear techniques single-photon emission tomography (SPECT) [16]. We have previously shown that T2DM is associated with myocardial microvascular abnormalities as evidenced by abnormal myocardial perfusion determined by quantitative RTMPE. We compared the accuracy of the RTMPE determinations with SPECT and found that a CFR cutoff of 1.9 provided sensitivity of 79%, specificity of 63%, and accuracy of 66% in T2DM patients [12].

In the current study, we sought to explore if there was a relationship between adequacy of T2DM control and abnormality of myocardial perfusion, using the noninvasive quantitative technique of RTMPE in patients with known or suspected CAD and referred for stress testing. We found that patients with poor control of their T2DM, defined as HgbA1c > 7.1%, had poorer myocardial microcirculatory perfusion, as evidenced by lower MBFR values. We also noted that this relationship was stronger in those patients that did not have established (known) CAD, but rather in those with known CAD.

Our findings are in alignment with the conclusions of the United Kingdom Prospective Diabetes Study (UKPDS) [17] which demonstrated that effective control of hyperglycemia can significantly reduce diabetic microvascular disease, especially when control is achieved early in the course of the diabetes. Similarly, a meta-analysis showed that lowering HgbA1c in T2DM decreased the risk of CHD and all-cause mortality. No specific thresholds were identified above which patients were at greater risk of developing CAD, but the greatest risk reduction was found in those with HgbA1c level below 7% [18]. However, there were still concerns regarding confounding factors affecting myocardial blood flow in T2DM subjects, such as age, gender, coexisting CAD, lipid disorders, smoking, and hypertension [19, 20]. In our study, after adjusting for those possible confounders, HgbA1c > 7.0% remained significantly associated with abnormal MBFR (defined as <2). We concluded that HgbA1c > 7.0% is an independent risk factor for having lower MBFR in T2DM patients.

Conversely, the ACCORD and VADT trials [21, 22] suggested that intensive diabetic control was disadvantageous in patients with long-standing diabetes and established CAD. Our results would support the findings in those studies as well, in that we observed that HgbA1c level had less apparent effect on the MBFR in those with established CAD, suggesting that when the microcirculation has been irreversibly affected by chronic glycemic elevations, there may be no benefit to achievement of tighter glycemic control.

Diabetes is a major risk factor for heart failure (HF) due to associated microvascular dysfunction. Recent studies [23, 24] have demonstrated that HgbA1c levels can predict HF hospitalization in T2DM patients, after adjusting for baseline cardiac and renal function. Moreover, HgbA1c has been shown to be an independent risk factor for HF regardless of the presence of coronary risk factors or development of coronary heart disease during follow-up. Thus, our results using RTMPE suggest the unique potential for serial utilization of a relatively inexpensive, portable, and noninvasive tool to assess microvascular function in T2DM receiving therapy targeted to HgbA1C level in order to prevent cardiac dysfunction.

Our study is limited primarily by the small numbers in our sample size. However, the findings are in accordance with observations from large clinical trials and suggest a potential mechanistic explanation for the dichotomous clinical interpretations of these trials. Our study suggests that further research using noninvasive techniques such as RTPME along with HgbA1c assessments may be useful to assess cardiovascular risk prediction in the growing T2DM population.

We also did not have concurrent angiographic evidence for the presence or absence of epicardial disease and relied on the history to establish a diagnosis of epicardial CAD. However, when we separated the patients into two groups by degree of glycemic control based upon HgbA1c levels, we did not find any significant differences in clinical characteristics or prior history of CAD.

At the current time, RTMPE is an off-label technique. However, it can be readily performed by using the same equipment and following the similar technical recommendations as currently approved for the on-label indication of left ventricular opacification and endocardial border enhancement during the assessment of left ventricular function.

## 5. Conclusions

Our findings are consistent with the published evidence that optimal glycemic control results in a lower incidence of abnormal microvascular perfusion. The observed inverse relationship between HgbA1c and MBFR in T2DM patients without known CAD suggests that improved glycemic control may reduce the likelihood of subsequent cardiovascular events. HgbA1c > 7.0% is an independent risk factor for having abnormal MBFR in T2DM patients. Early detection of and therapeutic intervention for vascular complications in T2DM patients are important to decrease the risk of CAD events. RTMPE provides a noninvasive, readily available opportunity for early detection of microvascular complications in T2DM patients, prior to development of clinical symptoms. In addition, the quantitative measurement of RTMPE used to assess microvascular function may be useful for future monitoring of therapeutic interventions in T2DM patients.

## Figures and Tables

**Table 1:** Clinical characteristics of the study population (*N* = 24).

Characteristics	Group 1 (*n* = 9) (HgbA1c > 7.1%)	Group 2 (*n* = 15) (HgbA1c ≤ 7.1%)	*P* value^a^
Age (years)	68.14 ± 3.91	64.34 ± 3.02	0.34
Males	7 (77)	9 (60)	0.36
BMI	31.38 ± 2.43	34.78 ± 1.88	0.15
Current smoking	5 (56)	5 (33)	0.28
Hypertension	7 (78)	13 (87)	0.58
Duration of known DM (years)	9.60 ± 2.52	8.0 ± 1.56	0.74
Known CAD	8 (89)	9 (60)	0.11
Dyslipidemia^b^	8 (89)	15 (100)	0.38
Previous MI	4 (44)	6 (40)	0.83
Previous CABG	5 (56)	6 (40)	0.46
Previous PCI	2 (22)	6 (40)	0.36
Medications			
Statins	8 (89)	12 (80)	0.56
ACE inhibitors	4 (44)	8 (53)	0.67
Aspirin	5 (56)	10 (67)	0.59
Beta Blocker	7 (78)	10 (67)	0.56
Calcium channels blockers	2 (22)	4 (27)	0.81
Nitrates	2 (22)	3 (20)	0.89
Rest			
HR (beats/min)	65 ± 7	70 ± 14	0.77
SBP (mmHg)	146 ± 17	135 ± 23	0.21
DBP (mmHg)	78 ± 9	69 ± 11	0.08
RPP (beats/min × mmHg)	9426 ± 951	9388 ± 2382	0.88
Adenosine stress			
HR (beats/min)	74 ± 12	80 ± 14	0.33
SBP (mmHg)	143 ± 8	124 ± 17	0.08
DBP (mmHg)	72 ± 12	65 ± 15	0.26
RPP (beats/min × mmHg)	10600 ± 1951	10086 ± 2142	0.44

BMI: body mass index; MI: myocardial infarct; CABG: coronary artery bypass graft; PCI: percutaneous coronary intervention; ACE: angiotensin converting enzyme; HR: heart rate; SBP: systolic blood pressure; DBP: diastolic blood pressure; RPP: rate-pressure product. Continuous variables were presented as mean ± standard deviation. Categorical variables were presented as numbers and percentages (%). ^a^Chi-square test for categorical data and independent *t*-test for continuous data comparison. ^b^Dyslipidemia was defined as total cholesterol (>210 mg/dl, or LDL > 130 mg/dl, or HDL < 35 mg/dl) or receiving lipid lowering medication.

**Table 2:** Comparison of quantitative myocardial perfusion parameters by segmental analysis.

	Group 1 (*n* = 107) (HgbA1c > 7.1%)	Group 2 (*n* = 165) (HgbA1c ≤ 7.1%)	*P* ^ g^ (rank sum test, 2- tail *P* value)
rBV_rest_	0.20 (0.15, 0.29)^a^	0.28 (0.21, 0.34)^d^	<0.001
rBV_stress_	0.28 (0.17, 0.38)	0.41 (0.30, 0.47)	<0.001
rBV_reserve_	1.39 (0.91, 1.78)	1.41 (1.08, 1.81)	0.51
*β*_rest_	5.12 (3.60, 9.52)^b^	6.16 (4.00, 8.92)^e^	0.33
*β*_stress_	4.51 (2.56, 18.46)	9.91 (3.91, 20.26)	0.004
*β*_reserve_	1.38 (0.71, 1.84)	1.87 (0.98, 2.49)	<0.001
MBF_rest_	1.05 (0.52, 1.91)^c^	1.62 (0.91, 2.57)^f^	0.002
MBF_stress_	1.20 (0.51, 4.80)	3.08 (1.31, 7.93)	<0.001
MBFR	1.71 (0.74, 3.22)	2.41 (1.31, 4.05)	0.004

rBV: rest relative blood volume; *β*: myocardial blood flow velocity; MBF: absolute myocardial blood flow; MBFR: myocardial blood flow reserve. Data is presented as median (25% IQR, 75% IQR). ^a,b,c^Wilcoxon sign rank test for comparison between baseline and stress perfusion parameters (rBV, *β*, and MBF) in HgbA1c > 7.1% group, *P* < 0.001. ^ d,e,f^Wilcoxon sign rank test for comparison between baseline and stress perfusion parameters (rBV, *β*, and MBF) in HgbA1c ≤ 7.1% group, *P* < 0.001. ^g^Wilcoxon rank sum test for comparison between HgbA1c > 7.1% group and HgbA1c ≤ 7.1% group.

**Table 3:** Univariate and Multivariate risk factors for abnormal MBFR < 2.

Variable	Odds ratio	95% confidence interval	*P* value	Odds ratio	95% confidence interval	*P* value
Sex	1.25	0.75, 2.06	0.39	—		
Age	1.02	1.00, 1.04	0.08	—		
Obesity BMI	1.13	0.66, 1.90	0.66	—		
Smoking status	1.54	0.95, 2.54	0.08	1.83	1.09, 3.12	0.02
Dyslipidemia	1.05	0.41, 2.73	0.92	—		
Duration of DM	1.12	0.69, 1.85	0.64	—		
Known CAD	1.21	0.74, 2.00	0.44	—		
HTN	1.0	0.98, 1.01	0.65	—		
High HgbA1c%	1.84	1.10, 3.11	0.02	1.92	1.12, 3.35	0.02

Obesity BMI defined as BMI > 30 and high HgbA1c% defined as >7.1%. Variables with likelihood test *P* value < 0.2 in univariate analysis were included in the multivariate logistic regression model and provided final adjusted odds ratios and 95% confidence intervals (95% CI).
